# WPD-Enhanced Deep Graph Contrastive Learning Data Fusion for Fault Diagnosis of Rolling Bearing

**DOI:** 10.3390/mi14071467

**Published:** 2023-07-21

**Authors:** Ruozhu Liu, Xingbing Wang, Anil Kumar, Bintao Sun, Yuqing Zhou

**Affiliations:** 1 School of International Education, Jiaxing Nanyang Polytechnic Institute, Jiaxing 314000, China; liuruozhu044@163.com; 2 College of Mechanical and Electrical Engineering, Wenzhou University, Wenzhou 325035, China; 20210129@wzu.edu.cn (A.K.); zhouyq@wzu.edu.cn (Y.Z.)

**Keywords:** rolling bearing, fault diagnosis, wavelet packet decomposition, graph convolutional neural network, deep graph contrastive learning

## Abstract

Rolling bearings are crucial mechanical components in the mechanical industry. Timely intervention and diagnosis of system faults are essential for reducing economic losses and ensuring product productivity. To further enhance the exploration of unlabeled time-series data and conduct a more comprehensive analysis of rolling bearing fault information, this paper proposes a fault diagnosis technique for rolling bearings based on graph node-level fault information extracted from 1D vibration signals. In this technique, 10 categories of 1D vibration signals from rolling bearings are sampled using a sliding window approach. The sampled data is then subjected to wavelet packet decomposition (WPD), and the wavelet energy from the final layer of the four-level WPD decomposition in each frequency band is used as the node feature. The weights of edges between nodes are calculated using the Pearson correlation coefficient (PCC) to construct a node graph that describes the feature information of rolling bearings under different health conditions. Data augmentation of the node graph in the dataset is performed by randomly adding nodes and edges. The graph convolutional neural network (GCN) is employed to encode the augmented node graph representation, and deep graph contrastive learning (DGCL) is utilized for the pre-training and classification of the node graph. Experimental results demonstrate that this method outperforms contrastive learning-based fault diagnosis methods for rolling bearings and enables rapid fault diagnosis, thus ensuring the normal operation of mechanical systems. The proposed WPDPCC-DGCL method offers two advantages: (1) the flexibility of wavelet packet decomposition in handling non-smooth vibration signals and combining it with the powerful multi-scale feature encoding capability of GCN for richer characterization of fault information, and (2) the construction of graph node-level fault samples to effectively capture underlying fault information. The experimental results demonstrate the superiority of this method in rolling bearing fault diagnosis over contrastive learning-based approaches, enabling fast and accurate fault diagnoses for rolling bearings and ensuring the normal operation of mechanical systems.

## 1. Introduction

With the rapid development of industrial production, the modern industry’s demand for machinery equipment is developing towards high quality, high intelligence, and high reliability. Rotating machinery is widely used in the chemical industry, mining, electric power, aviation, and other fields. Rolling bearing is a key component in the field of rotating machinery, and its failure will lead to serious economic losses and even casualties [1]. In order to ensure a safe and reliable production cycle, improve the production efficiency of enterprises, and provide effective intelligent diagnosis methods for the health status of rotor-bearing systems, it has become a hot research topic all over the world in recent years. Deep learning (DL) was used for fault diagnosis because traditional machine learning cannot meet the needs of contemporary industrial production. The fault diagnosis method based on DL further improves the intelligence of rotating machinery fault diagnosis technology with its powerful big data learning ability, nonlinear processing ability, and high generalization ability [2]. In recent years, the deep-learning-based fault diagnosis model has been widely studied and achieved excellent results [3,4,5].

In recent years, fault diagnosis methods based on DL have become a research hotspot around the world. DL models such as deep belief network (DBN), convolutional neural network (CNN), stacked auto-encoder (SAE), and generative adversarial neural network (GAN) are the most representative. For example, Li et al. [6] used Gaussian elements to construct Gaussian convolution DBN to realize the fault diagnosis of rotor-bearing systems under time-varying speeds. In order to solve the problem of low fault diagnosis efficiency under noise conditions, Xue et al. [7] improved CNN and proposed a new anti-noise CNN for fault diagnosis under noise background. Liu et al. [8] constructed two deep SAEs to extract features from the source domain and target domain of training data to solve the fault diagnosis in the adaptive environment of the partial domain. Tong et al. [9] proposed an auxiliary classifier GAN with spectral normalization for fault diagnosis with a small and unbalanced sample size. Huang et al. [10] decomposed the discrete vibration signals of gear boxes via wavelet packet, input the decomposed signal components into hierarchical CNNs, and adaptively extracted multi-scale features to effectively classify faults.

Although fault diagnosis methods based on DL have achieved good results in recent years [11], there are still some problems that seriously limit their application in practical production [12]. DL is mainly divided into supervised and unsupervised learning [13]. For supervised learning, obtaining a sufficient number of labeled fault samples is a challenging task. Moreover, dealing with the uneven distribution of various sample types, limited sample sizes, missing sample labels, and other issues related to data imbalance in fault data pose additional challenges. Particularly when working with unlabeled data, it becomes difficult to address these problems effectively using existing supervised intelligent fault diagnosis methods. As a result, the performance of the models significantly deteriorates, making it challenging to achieve high-precision intelligent fault diagnosis and monitoring for mechanical systems. Consequently, training the network to achieve satisfactory accuracy becomes increasingly difficult. Then, unsupervised learning can train the network to achieve satisfactory accuracy under the condition of unlabeled samples [14]. Therefore, self-supervised learning based on unlabeled data has received more and more attention and research from scholars. Wang et al. [15] proposed a one-stage self-supervised momentum contrastive learning model (OSSMCL) for open-set cross-domain fault diagnosis. The method is based on momentum encoders of self-supervised contrastive learning to capture distinguishable features between sample pairs and incorporates a one-stage framework of the meta-learning paradigm through which OSSMLC can learn to identify new faults with a small number of labeled samples in the target domain. Finally, the validity of the proposed method is verified on the open-set fault diagnosis dataset. An et al. [16] proposed a domain adaptation network (DACL) based on contrastive learning to realize the purpose of bearing fault diagnosis across different working conditions, reduce the probability of samples being classified near or on the boundaries of various types, and improve diagnosis accuracy. The proposed method consists of a feature mining module and an adversarial domain adaptation module. In the feature mining module, the one-dimensional convolutional neural network (1-D CNN) was used to extract the features of the original vibration signal. The adversarial domain adaptation module aims to learn domain-shared discriminative features for aligning marginal distributions. At the same time, a contrast estimation term is designed to quantify the similarity of data distribution, increase the distance between samples of different health states, reduce the probability of samples close to the boundary, and improve diagnostic performance. Finally, an adaptive factor was introduced to measure the relative importance of the method’s transfer ability and discrimination ability. The effectiveness of the proposed method is demonstrated in various fault diagnosis scenarios with domain differences between the source and target domains using experimental data from two bearing systems. Wang et al. [17] proposed a self-supervised contrastive learning framework based on nearest neighbor matching (SCLNNM) to solve the problem of limited labeled samples, which seriously affects the performance of fault diagnosis. The method proposed in this paper is used to learn discriminative feature representations from large-scale unlabeled data sets and then realize fault diagnosis. Since the collected mechanical 1D signals are different from 2D images, in addition to designing reasonable data augmentation combinations to generate similar real instances of 1D sequences, the proposed scheme also finds the nearest neighbors in the support set as positive instances of the input signals to increase the diversity of representations. In this framework, the 1D CNN model combined with contrastive learning aims to learn a robust generic representation from different augmented signals. Based on this, limited labeled data is finally used to investigate what kind of feature representation is appropriate and to train a simple classifier for fault diagnosis. The collected engine data set of an operating ship shows that the proposed framework can effectively extract valuable feature information and improve the classification accuracy under the limited labeled data set. Aiming at the problem of serious performance degradation of deep-learning fault diagnosis models caused by imbalanced data sets, Zhang et al. [18] proposed a new feature learning-based method named class-sensitive supervised contrastive learning (CA-SupCon). Supervised contrastive learning (Sup Con) is used for the first time in imbalanced fault diagnosis, which uses class information to optimize the feature differences between any two classes. In addition, a class-sensitive sampler (CA) is designed to rebalance the data distribution within each mini-batch during training, which improves Sup Con’s ability to expand the feature distance between any two minority fault states. By effectively integrating SupCon and CA, the proposed CA-SUPCON framework can obtain a more discriminative feature space with better intra-class compactness and inter-class separability and achieves good performance in the above class imbalance scenario. Extensive experiments on two open-source datasets demonstrate the effectiveness of the proposed method.

For rolling bearing, obtaining complete labeled data for single and compound faults is a very difficult and costly task, and how to solve the above problem from unlabeled data obtained from experiments or production has become a new topic in bearing-rotor system fault diagnosis [19]. GCL is a self-supervised learning algorithm for graph data, which aims to train a graph encoder on a given large amount of unlabeled graph data to obtain the feature representation vector of the graph [20]. The general process is similar to traditional CL, with the advantage of data augmentation of graph signals and contrast hierarchy enhancement [21]. The above advantages have been verified in the work of the literature [22,23,24,25]. The main work of this paper is as follows:

(1) We explored the distribution of wavelet energy in different frequency bands at the last level of wavelet packet decomposition for the original signal. Based on this, the Pearson correlation coefficient was introduced to calculate the correlation between wavelet energy in different frequency bands. Subsequently, a node graph construction method was proposed, where each frequency band served as a node, and the wavelet energy in the frequency band served as the node feature. The Pearson correlation coefficient was used as the edge weight between nodes, resulting in the construction of an undirected node graph to represent the information of the original signal.
(2) In consideration of the graph structure attributes of the node graph, the impact of node and edge deletion or addition on the graph structure and information was analyzed. Eventually, a method was proposed to use node and edge addition during the data augmentation phase. In the two augmentation steps, one involved computing the mean of the existing node features as the feature of the newly added node, while the other involved calculating the variance as the feature of the newly added node. The Pearson correlation coefficient was used to determine the relationship between the newly added node and the existing nodes, serving as the weight for the newly added edges.
(3) During the encoding process with graph convolutional neural networks, the weights of edges were utilized as the adjacency matrix, providing a more accurate representation of the relationships between the central node and its neighboring nodes.
(4) We analyzed the comprehensive performance of the proposed method using the vibration signal dataset from the bearing driving end of Western Reserve University. Experimental results demonstrate that WPDPCC-DGCL exhibits superior data processing capability and achieves better fault diagnosis of rolling bearings compared to contrastive learning (CL).

The remainder of this article is arranged as follows: the second section mainly introduces the proposed WPDPCC-DGCL method, the third section verifies the feasibility of the proposed method using the bearing data of Western Reserve University, and the fourth section summarizes the main achievements made in this paper.

## 2. Proposed Method

The proposed WPDPCC-DGCL method in this paper first performs sliding window sampling on the collected 1D vibration signal data, followed by four-level WPD processing of the sampled window values. Each frequency band in the last layer of decomposition is considered a node, and the energy values on each frequency band are used as node features to construct the node graph, which performs two data augmentation on the node graph to obtain $G_{1}$ and $G_{2}$, uses the feature vector Q and adjacency matrix $\partial_{ij}$ of the sample graph after data augmentation as the input of the GCN, and uses the GCN to the coded representation, which is used to obtain a more complete feature vector y. The new feature vectors $z_{i}$ and $z_{j}$ are obtained by mapping y to a low-dimensional space by connecting MLP after GCN, training the pre-training model based on DGCL, and using the weight values extracted from the features in pre-training as the initial values for feature extraction in the classification model.

### 2.1. WPDPCC Construct Node Graphs

#### 2.1.1. Vibration Signal WPD Stage

WPD is a modern time–frequency analysis that could effectively process all kinds of non-stationary random signals. In the past, it was common to use WPD simply as a tool for feature extraction [26,27]. However, it is worth noting that the proposed method is an improvement upon wavelet decomposition. It decomposes both the high-frequency and low-frequency components of the signal, resulting in a finer and more comprehensive decomposition than traditional wavelet transform. This approach effectively captures the full-frequency characteristics of the signal. The feature vectors obtained from this decomposition can adaptively select frequency bands with time–frequency localization characteristics, enabling improved time–frequency resolution of the signal. Consequently, WPD demonstrates a strong capability in processing nonstationary signals [28].

**Figure 1:**
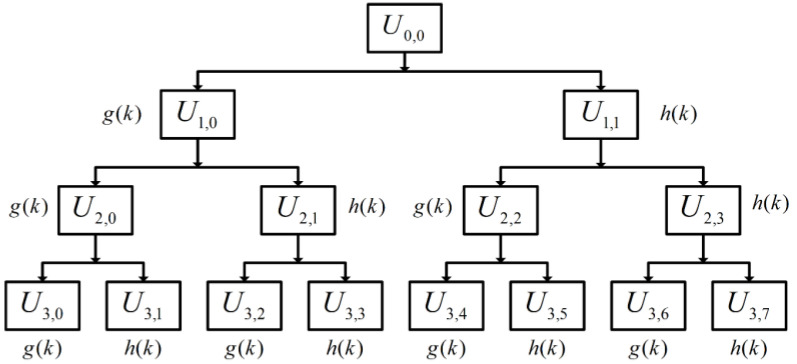
Signal time–frequency space division diagram with WPD.

At this stage, the original signal can be decomposed into low frequency and high frequency using WPD, and the original discrete vibration signal $U_{0,0}$ can be decomposed into its signal morphology in different frequency bands v WPD. Taking three-layer WPD as an example, its structure is shown in Figure 1.

$U_{i,j}$ represents the decomposed signal corresponding to the *j*-th node of the *i*-th layer (scale number). Decomposed signals calculated at different decomposition layers can be calculated layer by layer in (1) and (2) as follows:

(1) $$U_{i+1,2j}\left(n\right)=\sum_{k}g(k-2n)U_{i,j}(k)$$

(2) $$U_{i+1,2j+1}\left(n\right)=\sum_{k}g(k-2n)U_{i,j}(k)$$

where k is the discrete-time series, n is the time shifting factor, $g\left(k\right)$ is low-pass filter coefficient, and h(k) is high-pass filter coefficient. When the node number *j* is even, it represents the low-frequency component signal obtained via low-pass filtering coefficient $g\left(k\right)$ decomposition; when *j* is odd, it represents the high-frequency component signal obtained via high-pass filtering coefficient h(k) decomposition.

High-pass and low-pass filtering coefficients need to satisfy the orthogonal relationship was computed in (3) as follows:

(3) $$g(k)={\left(-1\right)}^{k}h(1-k)$$

The original signal is decomposed using WPD into signal components at different feature scales (frequency bands), which is equivalent to passing the original signal through a series of filters with different center frequencies but the same bandwidth [29]. It is assumed that X is the original signal collected by the sensor, and X is continuously sampled by a non-overlapping sliding window with a length of 1024 data points to obtain multiple continuous signal segments as $\left\{X^{1},X^{2},X^{3},\ldots X^{n},\ldots \right\}$. Then, WPD is carried out on the signal $X^{n}$ to extract the feature vectors of signal components under different feature scales in the last layer of signal $X^{n}$. According to the needs of node graph construction, In the last layer, the wavelet energy [30] of each frequency band after WPD is used to form the feature vector for each node $q_{i}$ in (4).

(4) $$q_{i}=\left[E_{i}\right]$$

where *i* denotes the sequence of frequency band, $E_{i}$ is the wavelet energy that $q_{i}$ denotes the feature of the *i*-th frequency band of the original signal at the last layer. Wavelet energy could be calculated in (5).

(5) $$E\left(j,i\right)=\sum_{k\in Z}{\left[p_{s}\left(n,j,k\right)\right]}^{2}$$

where $E\left(j,i\right)$ denotes the energy value of the *i*th node on the decomposition layer j; $p_{s}\left(n,j,k\right)$ is the wavelet packet coefficient. In practice, the energy of each node is often normalized to take the percentage of the energy of each node. So, the characteristics of the original signal can be expressed in (6).

(6) $$Q={\left[q_{0},q_{1},q_{2},\ldots ,q_{15}\right]}^{T}$$

Compared with feature extraction directly from the original signal, WPD can extract features from the signal components of different frequency bands more efficiently. By considering the features of each frequency band as node features, Q can be regarded as a feature vector of a node graph.

**Figure 2:**
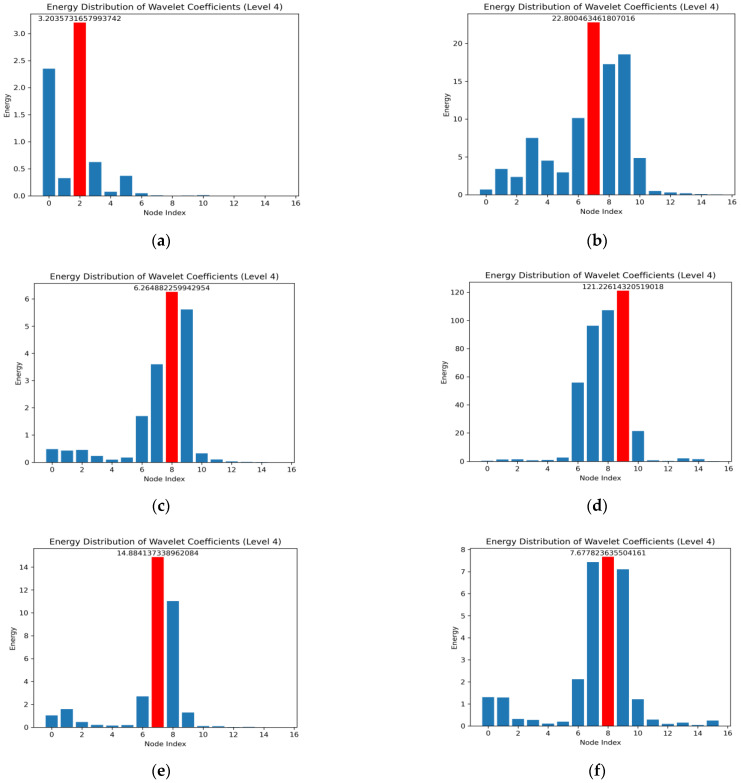

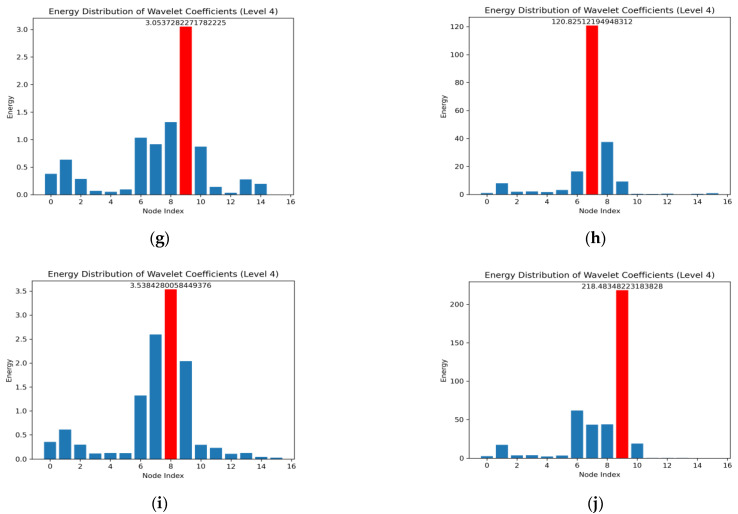
Wavelet energy diagram on each type of fault node: (**a**) Normal condition; (**b**) Inner ring failure under 0.007-inch damage; (**c**) Outer ring failure under 0.007-inch damage; (**d**) Rolling body failure under 0.007-inch damage; (**e**) Inner ring failure under 0.014-inch damage; (**f**) Outer ring failure under 0.014-inch damage; (**g**) Rolling body failure under 0.014-inch damage; (**h**) Inner ring failure under 0.021-inch damage; (**i**) Outer ring failure under 0.021-inch damage; (**j**) Rolling body failure under 0.021-inch damage.

According to the processing of the original signal, the 10 types of fault signals are decomposed into four layers with db6 as the wavelet basis function, and the wavelet energy diagram on each type of fault node is shown in Figure 2. Analysis of the wavelet energy diagram shows that the second node has the highest energy under normal conditions, the seventh node has the highest energy under an inner ring fault, the eighth node has the highest energy under an outer ring fault, and the ninth node has the highest energy under a rolling body fault.

#### 2.1.2. Constructing Node Graph Stage

Graph data are capable of effectively representing the correlation between data objects [31]. Hence, in this stage, the process of modeling the feature vector for each signal component after WPD decomposition is introduced using an undirected weighted graph. The graph data is composed of a group of nodes and edges, and the overall representation of the graph is constructed by constructing the relationship between nodes and edges. PCC [32] is used to calculate the weight between the feature vectors of each signal component and construct edges, and WPDPCC method proposed in this paper is used to construct node graph, which has two obvious advantages: firstly, the sample graph can represent the feature vector of each signal component as a whole from a global perspective; secondly, the weights between nodes are utilized to denote the relationships between the feature vectors. PCC is used to measure the magnitude of the linear correlation between two variables, *X* and *Y*, and its value is between −1 and 1. For the value of PCC between two nodes, it is calculated in (7), (8), and (9) as follows:

(7) $$\bar{X}=\frac{\sum_{i=1}^{n}X_{i}}{n},\;\bar{Y}=\frac{\sum_{i=1}^{n}Y_{i}}{n}$$

(8) $$Cov\left(X,Y\right)=\frac{\sum_{i=1}^{n}\left(X_{i}-\bar{X})(Y_{i}-\bar{Y}\right)}{n-1}$$

(9) $$r_{XY}=\frac{Cov(X,Y)}{S_{X}S_{Y}}$$

where $r_{XY}$ denotes the value of the sample PCC, $Cov\left(X,Y\right)$ denotes the sample covariance, $S_{X}$ denotes the sample standard deviation of node *X*, and similarly, $S_{Y}$ denotes the sample standard deviation of node *Y*, which computed in (10) and (11) as follows:

(10) $$S_{X}=\sqrt{\frac{\sum_{i=1}^{n}{\left(X_{i}-\bar{X}\right)}^{2}}{n-1}}$$

(11) $$S_{Y}=\sqrt{\frac{\sum_{i=1}^{n}{\left(Y_{i}-\bar{Y}\right)}^{2}}{n-1}}$$

Therefore, the matrix consisting of Pearson correlation coefficients between nodes in each fault sample diagram in (12) is expressed as follows:

(12) $$\rho =\left[\begin{array}{ccc} r_{00} & \cdots & r_{15}\\ \vdots & \ddots & \vdots \\ r_{15} & \cdots & r_{15} \end{array}\right]$$

The graph data can be generally expressed as G={V, E}, where *V* denotes the set of nodes, and *E* is the set of edges, and the signal components of wavelet coefficient are treated as nodes, and all nodes in the third layer constitute the vertex set *V* of *G*. The relationship between each node is determined via PCC, and if it is correlated, it means that there is an edge relationship between two nodes, and the value of PCC is saved on the graph as the weight of the edge, and if it is not correlated, it means that there is no edge relationship between two nodes. The process of constructing a sample graph from the vertices and PCCs in the absolute *V* is as follows:

1. Considering the feature vector $Q_{i}$ of each frequency band as a vertex $V_{i}$ of a nodal graph;
2. Calculating the PCC between two vertices and using the value of PCC as the weight of the edge between the two nodes;
3. Constructing the adjacency matrix $\partial_{ij}$ of the sample graph g, which represents the relationship of the edges between each vertex, where $\partial_{ij}$ is a symmetric matrix, and take the lower triangle for convenience of subsequent calculations.

**Figure 3:**
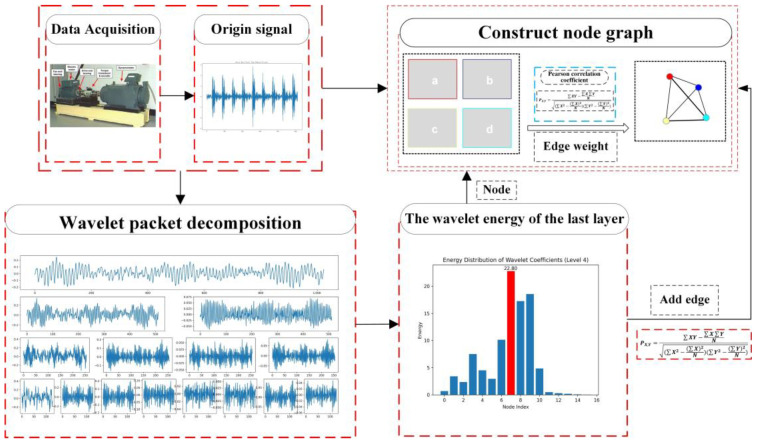
Process of constructing nodal graph method based on WPD-PCC.

According to the above process, the constructed sample diagram can be put in Figure 3. The study of using WPDPCC to construct sample diagrams in rotor-bearing system fault diagnosis is detailed in Section 3 of this paper.

### 2.2. DGCL Pre-Training Model

#### 2.2.1. Data Augmentation Stage

Data augmentation is a common means of data processing in the field of machine learning, generally by cropping, rotating, and other operations on images to produce enhanced data. However, such methods are poor at processing node graph data, as illustrated in paper [33], which argues that traditional geometric transformations for data augmentation are not universally applicable to graph structures. Therefore, for different categories of graph structures, it is necessary to explore specific data augmentation methods tailored to graph structures. Consequently, the authors propose four general data augmentation methods for graph structures, namely node dropping, edge perturbation, attribute masking, and subgraph. On the other hand, data augmentation is a prerequisite for DGCL and is considered the process of transforming and generating new data via reasonable transformations. It aims to retain the information of the original data to some extent and achieve the purpose of enhancing the robustness of the dataset.

According to the characteristics of node graph, this paper employs the augmentation technique of adding edges and nodes to enhance the node graph data. The criteria for adding nodes were based on the mean and variance of node features within the node graph. In the first augmentation step, we added one node with the mean, serving as the node feature. Subsequently, in the second step, we added another node with the variance as the node feature. To establish connections between the newly added nodes and existing nodes, we utilized the PCC as a measure so that the input image G produces two different node graphs $G_{1}$ and $G_{2}$ with positive sample pairs.

#### 2.2.2. Node Graph Feature Learning Stage

In this stage, the constructed sample graph is encoded using graph attention networks to represent the graph [34]. GCN mainly employs message passing and aggregation mechanisms for node graph feature extraction, and aggregation methods are used to average the information of neighboring nodes. Specifically, GCN is divided into spectral-domain GCN and spatial-domain GCN. The spectral-domain GCN maps the nodes to the frequency-domain space through the Fourier transform (used to connect the null and frequency domains), achieves convolution in the time domain through convolution on the frequency domain, and finally maps the features back to the null domain. When the Fourier change is not available, the spectral domain GCN will also fail. In addition, the spectral domain GCN cannot make the graph structure change during training, i.e., it cannot remove nodes and edges, so it cannot satisfy the data enhancement during DGCL pre-training. In contrast, the spatial domain GCN redefines convolution on the graph by passing the spectral graph theoretical convolution and can directly define the convolution operation on the space and perform convolutional feature extraction based on nodes and neighbors directly [35].

According to the fault sample graphs constructed in the previous stage, each sample graph has 8 nodes, and the feature vector of each node is $q_{i}=\left[E_{i}\right]$. Then, the dimension of the feature vector Q of each graph is 16 × 1, and the dimension of the adjacency matrix $\partial_{ij}$ between each node is 16 × 16. q and $\partial_{ij}$ are the inputs of the GCN model.

For each graph, the propagation between each layer in the GCN in (13) is calculated as follows:

(13) $$Q^{\left(l+1\right)}=\sigma \left({\tilde{D}}^{-\frac{1}{2}}\tilde{A}{\tilde{D}}^{-\frac{1}{2}}Q^{\left(l\right)}W^{\left(l\right)}\right)$$

where σ is the nonlinear activation function ReLU, Q is the eigenvector matrix of each graph in each layer, $W^{\left(l\right)}$ is the trainable parameter matrix of the convolution transform of the current layer, and $\tilde{D}$ is the degree matrix of $\tilde{A}$ computed in (14) as follows:

(14) $$\tilde{D}=\sum {\tilde{A}}_{ij}$$

where $\tilde{A}=A+I$, I is the unit matrix, the node itself is considered, and in order to consider the phenomenon that the feature vectors are continuously summed up when aggregating the features of the neighboring nodes of a node, which leads to a larger feature of the node with more neighboring nodes, symmetric normalization is used in this paper. Constructing a two-layer GCN with ReLU and Softmax as activation functions, the forward propagation formula of the GCN can be expressed in (15) as follows:

(15) $$y=f\left(Q,A\right)=softmax(\tilde{A}ReLU(\tilde{A}QW^{\left(0\right)})W^{\left(1\right)})$$

Therefore, the input feature vector matrix *Q* can be obtained as the new feature vector matrix *y* after the propagation of the above two-layer GCN, and it is used as the input of the DGCL pre-training model.

#### 2.2.3. Projection Head Stage

This stage focuses on setting the mapping layer network structure $g\left(\cdot \right)$ after the GCN network model to map the GCN-encoded feature vectors to the low-dimensional space to obtain the low-dimensional feature vectors for the calculation of the contrast loss function. Here, a three layers multilayer perceptron (MLP) is chosen and normalized after each linear layer. So, the output y of the previous stage is characterized to obtain $z_{i}$ and $z_{j}$ can be expressed in (16) as follows:

(16) $$z_{i}=g\left(y\right)=W^{\left(2\right)}\sigma \left(W^{\left(1\right)}y\right)$$

where σ is the ReLU nonlinear activation function.

#### 2.2.4. Contrast Loss Function

The *N* images obtained by random sampling at this stage are used as a training batch, and according to the above process, it is known that each sample in each batch will undergo two data enhancements and produce 2N data points, and the remaining 2(N−1) samples after a given positive sample pair are used as negative samples. A contrast loss function $l_{i,j}$ is defined to achieve a consistent type maximization of positive sample pairs compared with negative samples, and, here, the normalized temperature scale cross-entropy loss is utilized [36]. Therefore, the contrast loss function can be defined in (17) as follows:

(17) $$l_{i,j}=-log\frac{exp\left(sim\left(z_{i},z_{i}\right)/\tau \right)}{\sum_{k=1}^{2N}F_{\left[k\neq i\right]}exp\left(sim\left(z_{i},z_{k}\right)/\tau \right)}$$

This loss function has been widely used in sub-supervised learning research in recent years. $F_{\left[k\neq i\right]}\in \left\{0,1\right\}$, the value of this indicator, is 1 when k≠i, τ is the temperature coefficient, τ = 0.1 in this paper, and the cross loss is calculated between all positive samples in a training batch. $sim\left(z_{i},z_{i}\right)$ refers to the cosine similarity, which is calculated in (18) as follows:

(18) $$sim\left(z_{i},z_{i}\right)=\frac{{z_{i}}^{T}z_{j}}{\left\|z_{i}\|\|z_{j}\right\|}$$

Finally, all losses in n batches are calculated via Equation (17) and the average value is taken to obtain the final loss *L* computed in (19) as follows:

(19) $$L=\frac{1}{2N}\sum_{k=1}^{2N}\left[l\left(2k-1,2k\right)+l\left(2k,2k-1\right)\right]$$

#### 2.2.5. Fault Diagnosis Procedure

**Figure 4:**
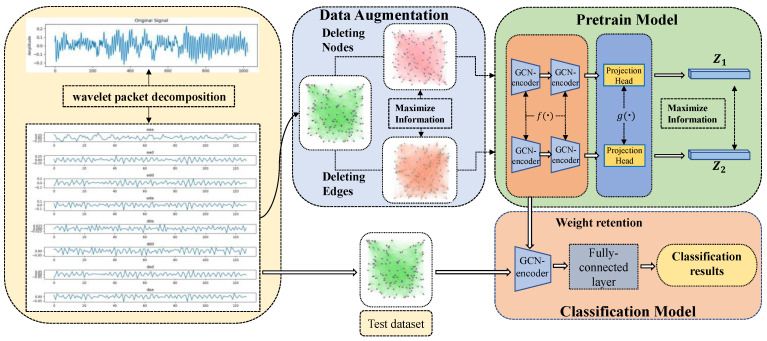
Rolling bearing fault diagnosis process based DGCL method.

The flow of the proposed rolling bearing fault diagnosis method based on WPDPCC-DGCL is depicted in Figure 4. The fault diagnosis process consists of two steps. The first step is the training phase, where the objective is to obtain a well-trained model. The second step is the testing phase, which involves classifying the fault node graph using the DGCL model.

## 3. Case Study

In this section, we evaluate the proposed WPDPCC-DGCL method using vibration signals from the shaft end of the Case Western Reserve University (CWRU) bearing dataset and bearing data from the University of Paderborn in Germany as the raw data.

### 3.1. The Performance on the CWRU Dataset

#### 3.1.1. Data Sources

**Figure 5:**
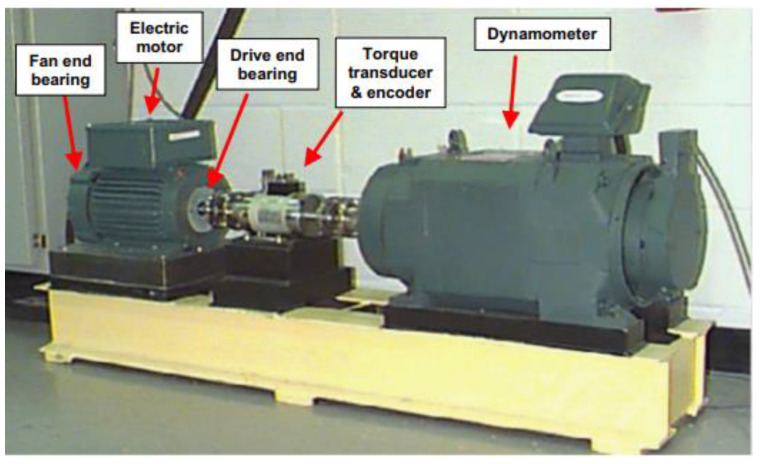
CWRU bearing test stand [37].

The vibration signal dataset used in this paper is the bearing failure benchmark dataset published by the CWRU Data Center [37]. The test stand used to collect the bearing defect detection signal is shown in Figure 5, which consists of a 1.5 W motor, torque sensor decoder, and a power test meter. A damage of 0.007–0.040 inches in diameter was caused via EDM at the rolling element, inner ring, and outer ring locations of the bearing, and was mounted at the drive end and fan end of the test stand, respectively, with the bearing model numbers SKF6205 and SKF6203 at the two locations, and vibration signals were recorded for loads ranging from 0 to 3 hp (motor speed of 1797-1720 RPM).

#### 3.1.2. Parameter Setting

The data used in this paper is the model SKF6205 with the following properties, as shown in Table 1: the motor speed of 1772 rpm; load for 1 horsepower; sampling frequency of 12 Khz drive end of the vibration signal generated, respectively; in the bearing failure diameter of 0.007 inches, 0.014 inches, and 0.021 inches, respectively; selected bearing rolling body; inner ring, outer ring (selected 6 o’clock direction of the fault) vibration signals; a total of 9 types of vibration signals with faults, in addition to a class of normal vibration signals; a total of 10 types of vibration signals constitute the data set.

In the original dataset, there are 112,571 data points in each class of the vibration signal of the fault. In this paper, we use the sliding window sampling method to make 1024 data points in each class of the vibration signal into one sample, and 600 samples are collected in each class, so the dataset contains a total of 6000 samples. The labeled data set is randomly divided equally into a training set and a test set, and the training set and the unlabelled data set are used to form a new training set for training the DGCL pre-training model. Finally, 30 samples from each class of the test set were selected as the validation set, and the remaining 30 samples were used as the test set. A total of 30, 50, 70, and 90 node graphs were selected from each class of the pre-trained model and the previous test set to form a new test set BR-30, BR-50, BR-70, and BR-90, respectively.

#### 3.1.3. Results and Analysis

The original node graph and the node graphs obtained after two rounds of data augmentation are used as positive sample pairs, and the rest of the node graphs are used as negative samples for pre-training the DGCL model, the parameters of the pre-trained model are shown in Table 2.

**Figure 6:**
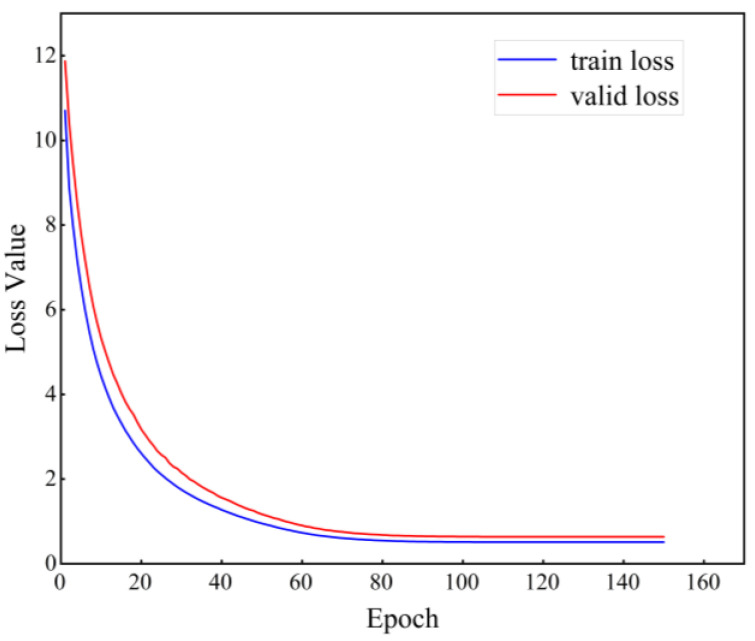
The change in pre-training contrastive loss function with 150 epochs.

The optimal parameters of the model are saved during the pre-train, which obtains satisfactory results and the loss function of the training as Figure 6.

**Figure 7:**
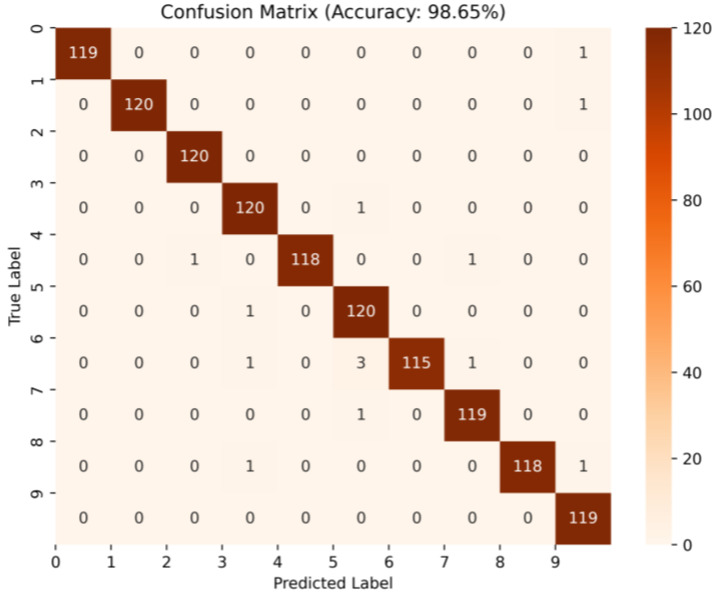
The confusion matrix under the BR-90 test set.

By averaging the results of five replicate experiments of the three methods, Table 3 shows that when there are 90 node graphs per category in the training set, the test accuracy of each category in the test set reaches 98.65%, and the confusion matrix is shown in Figure 7. Compared with the other three methods, the test accuracy of this method has improved by 9.29%. When the training set in the test set had only 30 node graphs per category, the dataset classification results obtained with WPDPCC-DGCLL also had a test accuracy class of 80.69%, which was much higher than the other methods. It can be seen that the advantage of the method is more obvious when the sample size is larger.

### 3.2. The Performance on the Paderborn University Dataset

#### 3.2.1. Data Sources

**Figure 8:**
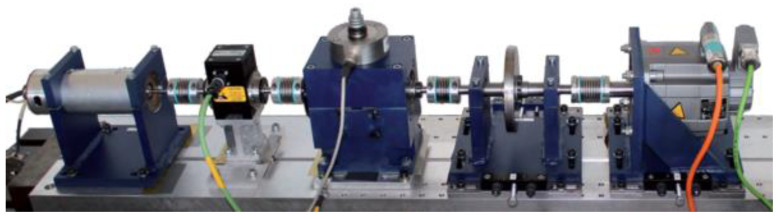
The German Paderborn University bearing test stand.

The German Paderborn University Bearing Dataset provides a collection of experimental bearing data for condition monitoring based on vibration and motor current signals. The test rig is a modular system capable of generating the necessary measurement data for analyzing the corresponding features and damage characteristics obtained from motor current signals. The basic components of the test rig include a drive motor (permanent magnet synchronous motor) acting as a sensor, a torque measurement shaft, a test module, and a load motor. The test stand used to collect the bearing defect detection signal is shown in Figure 8.

The dataset includes artificially induced and real damages. Vibration signals from the bearing housing are collected using piezoelectric accelerometers, with a sampling frequency of 64 kHz. The operating conditions are varied by changing the rotational speed of the drive system, applying radial forces on the bearing, and adjusting the load torque on the drive system during the sampling process. In the dataset, high-resolution vibration data was collected from six healthy bearings and 26 sets of faulty bearings. Among the 26 sets of faulty bearings, 12 were artificially damaged, and 14 were subjected to accelerated life testing to simulate real damage. We selected vibration data from three healthy bearings and six bearings that were damaged using accelerated life testing. Among these six faulty bearings, half had inner race faults, and the other half had outer race faults. The accelerated life testing for the bearings used in this study was conducted at a rotational speed of 1500 RPM, a torque load of 0.7 Nm, and a radial force of 1000 N. Experiments with three healthy bearings and a different time of operation were performed as reference states, as shown in Table 4. The details of each of the six faulty bearings considered in the diagnosis problem, as shown in Table 5.

#### 3.2.2. Data Preparation

To ensure an adequate sample size, we performed sliding window sampling on the vibration signals in the X direction for each state. The window size was set to 2048, with 100 samples per file and a step size of 118. Since each state had 20 sets of X-directional data, the number of samples per state was 100 ∗ 20 = 2000. Therefore, the total number of samples in the entire dataset was 2000 ∗ 9 = 18,000. We used the WPD-PCC method to construct 18,000 node graphs based on the sliding window samples. The dataset was then divided into a training set (14,400 samples), test set (1800 samples), and validation set (1800 samples) in an 8:1:1 ratio. In the training set, all node graphs do not have any class labels assigned to them. However, in the test set, each node graph is associated with a specific class label. From the test set, 100, 250, 400, and 500 node graphs are selected without their corresponding class labels. These node graphs, along with the training set, will constitute the training set for pre-training the model. These subsets of the test set are defined as BR100, BR250, BR400, and BR500, respectively. These node graphs that are included in the pre-training process will still be used in the downstream classification task.

#### 3.2.3. Classification Results

The original node graph and the node graph after data enhancement are used as positive sample pairs, and the rest of the node graphs are used as negative samples for pre-training the DGCL model, the parameters of the pre-trained model are shown in Table 6.

**Figure 9:**
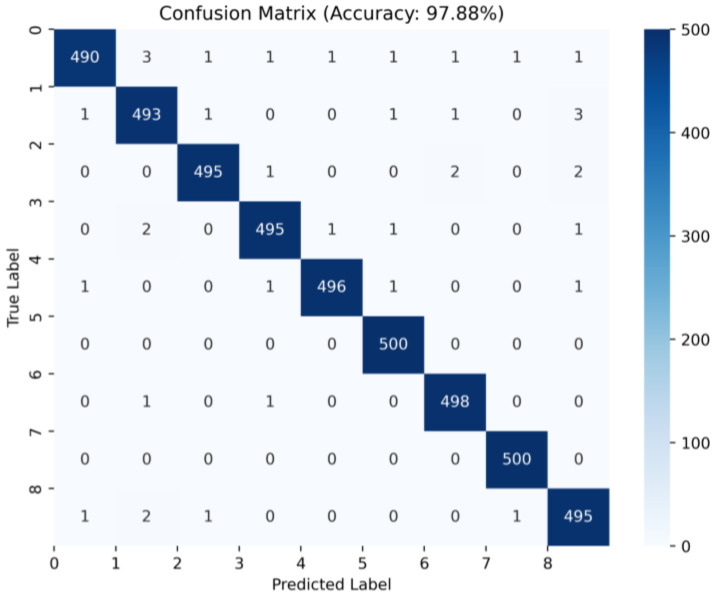
The confusion matrix when the test set is BR500.

Through five replicate experiments and averaging the results of three methods, Table 7 shows that the test accuracy reached 97.88% when the training set contained 500 node graphs for each category in the test set. Compared to the other two methods, this method achieved an 8.45% improvement in accuracy. When the training set contained 100 node maps for each category in the test set, the dataset obtained using the WPDPCC-DGCLL method also achieved a test accuracy of 75.63%, significantly higher than the other methods. It can be observed that this method has a more pronounced advantage with larger sample sizes. The confusion matrix when the test set is BR500 is shown in Figure 9.

## 4. Conclusions

In this paper, we propose a fault diagnosis method for rolling bearings based on WPDPCC-DGCL, which focuses on extracting signal component information of different frequency bands from the unlabeled data of rolling bearing time series. The main contribution of the method is to propose the WPDPCC method of constructing node graphs to build the dataset and pre-training it on the DGCL model, to combine the advantages of node graphs with data enhancement by randomly removing nodes and edges, which provides a more complete information representation of graph data in space compared to the one-dimensional time-domain data, and to explore the application of the DGCL method in downstream tasks in the fault diagnosis domain. The results show that the self-supervised pre-training model is effective compared to the traditional method knot in the case of large amounts of unlabeled data. However, there are several issues that need to be addressed as follows:

(1) High requirements for pre-processing of the original signal and the need for comprehensive analysis in conjunction with the characteristics of the original signal in the construction of a high-quality node graph;
(2) The long training time of the DCCL method due to the large amount of data and the repetition of positive and negative samples during the training process;
(3) The generalization capability of the model needs to be improved, and the mode of data set processing needs to be modified in the future.

In order to better solve the above problems, in the future, the research on fault diagnosis based on DGCL can be improved from the perspective of data acquisition, the method of constructing node graphs and data augmentation, and the specific analysis is as follows:

(1) Considering the spatial layout of sensors in the initial stage, data preprocessing is used to decompose the 1D time series data at different spatial locations, and the results of 1D signal decomposition are concatenated on the spatial layout according to the location of sensors to achieve the multi-dimensional representation of the signal;
(2) Keeping up exploring the signal decomposition methods, such as wavelet packet decomposition, empirical mode decomposition, and other methods in the application of 1D signal decomposition, extracting more accurate and complete feature vectors as node features, and determining the weight relationship between nodes by measuring the distribution similarity and distance of node features in space to provide a feasible theoretical method for constructing high-quality node graphs;
(3) The experimental verification of data enhancement methods of deleting nodes or adding nodes is improved to ensure the interpretability and feasibility of data enhancement.

## Figures and Tables

**Table 1:** Bearing failure classification and failure information.

Category	File Name	Failure Location	Size (Inch)
0	97.mat	normal	0
1	106.mat	inner race	0.007
2	131.mat	out race	0.007
3	119.mat	ball	0.014
4	170.mat	inner race	0.014
5	198.mat	out race	0.014
6	186.mat	ball	0.021
7	210.mat	inner race	0.021
8	235.mat	out race	0.021
9	223.mat	ball	0.021

**Table 2:** Pre-training parameters for CWRU bearing dataset.

Epoch	In Channels	Hidden Channels	Out Channels	Batch Size
150	1	32	10	32

**Table 3:** Classification results of three methods for CWRU bearing dataset under different sample sizes.

Method	BR-30	BR-50	BR-70	BR-90
WPDPCC-GCN	62.30%	65.23%	71.51%	79.65%
WPDPCC-CL	71.35%	74.50%	79.68%	89.36%
WPDPCC-DGCL	80.69%	84.72%	92.31%	98.65%

**Table 4:** Operating parameter of healthy (undamaged) bearings during run-in period.

Bearing Code	Run-in Period [h]	Radial Load [N]	Speed [min^−1^ ]
K001	>50	1000–3000	1500–2000
K002	19	3000	2900
K004	5	3000	3000

**Table 5:** Test bearings with real damages caused by accelerated lifetime tests.

Bearing Code	Bearing Name	Damage	Class	Combination	Arrangement	Damage Extent	Characteristic of Damage
KA04	OR1	fatigue: pitting	OR	S	no repetition	1	single point
KA16	OR3	fatigue: pitting	OR	R	random	2	single point
KA22	OR4	fatigue: pitting	OR	S	no repetition	1	single point
KI04	IR1	fatigue: pitting	IR	M	no repetition	1	single point
KI14	IR2	fatigue: pitting	IR	M	no repetition	1	single point
KI16	IR3	fatigue: pitting	IR	S	no repetition	3	single point

Note: IR—Inner Race Defect; OR—Outer Race Defect; S—Single Damage; R—Repetitive Damage; M—Multiple Damage.

**Table 6:** Pre-training parameters for German Paderborn University Bearing Dataset.

Epoch	In Channels	Hidden Channels	Out Channels	Batch Size
150	1	64	32	64

**Table 7:** Classification results of three methods for German Paderborn University Bearing Dataset under different sample sizes.

Method	BR-100	BR-250	BR-400	BR-500
WPDPCC-GCN	61.25%	65.19%	70.96%	78.75%
WPDPCC-CL	70.29%	75.32%	80.58%	89.43%
WPDPCC-DGCL	75.63%	83.49%	91.84%	97.88%

## Data Availability

This study did not report any data.
